# Changing the Tune: Listeners Like Music that Expresses a Contrasting Emotion

**DOI:** 10.3389/fpsyg.2012.00574

**Published:** 2012-12-24

**Authors:** E. Glenn Schellenberg, Kathleen A. Corrigall, Olivia Ladinig, David Huron

**Affiliations:** ^1^Department of Psychology, University of Toronto MississaugaMississauga, ON, Canada; ^2^School of Music, Ohio State UniversityColumbus, OH, USA

**Keywords:** music, emotion, liking music, music preferences, contrast effect, hedonic ratings

## Abstract

Theories of esthetic appreciation propose that (1) a stimulus is liked because it is expected or familiar, (2) a stimulus is liked most when it is neither too familiar nor too novel, or (3) a novel stimulus is liked because it elicits an intensified emotional response. We tested the third hypothesis by examining liking for music as a function of whether the emotion it expressed contrasted with the emotion expressed by music heard previously. Stimuli were 30-s happy- or sad-sounding excerpts from recordings of classical piano music. On each trial, listeners heard a different excerpt and made liking and emotion-intensity ratings. The emotional character of consecutive excerpts was repeated with varying frequencies, followed by an excerpt that expressed a contrasting emotion. As the number of presentations of the background emotion increased, liking and intensity ratings became lower compared to those for the contrasting emotion. Consequently, when the emotional character of the music was relatively novel, listeners’ responses intensified and their appreciation increased.

## Introduction

A stimulus is perceived differently depending on whether it is presented in isolation or in context. In vision, for example, the same gray square looks lighter or darker depending on whether it is presented against a black or white background, respectively (i.e., White’s, 2010 illusion). In audition, the same tone is perceived to sound louder or softer when it is presented among softer or louder background tones, respectively (e.g., Melamed and Thurlow, 1971). Similarly, the temperature of the same tactile stimulus appears to increase or decrease after exposure to a relatively cold or warm stimulus, respectively (Locke, 2008). In general, then, the perceived magnitude of a stimulus along some continuous parameter (e.g., lightness, loudness, and temperature) shifts such that it is further from stimuli presented in the same context, a phenomenon known as the *contrast effect*. This phenomenon extends to higher-level evaluative processes, or *hedonic contrasts* (Parducci, 1995). For example, evaluations of pieces of music increase or decrease depending on whether previously heard pieces sounded bad or good, respectively (Parker et al., 2008). Similar hedonic-contrast effects are observed with tastes (Zellner et al., 2003), pictures of birds (Zellner et al., 2003), paintings (Dolese et al., 2005; Zellner et al., 2010), and the degree to which people are considered physically attractive (Kenrick and Gutierres, 1980).

Hedonic contrasts are especially relevant for responses to works of art and other stimuli that are evaluated esthetically. Emotional responding to art differs from responding to other stimuli because it occurs on two levels: one related to the emotion expressed by the work of art, the other to the perceiver’s evaluation (Hunter and Schellenberg, 2010). Accordingly, perceivers can have a positive evaluation of a stimulus that expresses a negative emotion, such as when they like sad-sounding music (e.g., The Beatles’ *Yesterday*) or paintings that portray distress (e.g., Munch’s *The Scream*). Positive hedonic evaluations are important psychologically because they can lead to perceptual sensitization (Vanderplas and Blake, 1949). For example, when presented at a low amplitude, words are identified more successfully if they are evaluated favorably rather than unfavorably. In the case of music, pieces that are positively evaluated are remembered better than pieces with neutral or negative evaluations (Stalinski and Schellenberg, 2012).

In the present study, we were interested in emotional responses to esthetic stimuli – those that pose no immediate threat or benefit to survival. Our specific focus was on liking music, and how listeners evaluate excerpts of music as a function of whether the emotion they express contrasts with the emotion expressed by music heard previously. Theories about the psychology of esthetics speculate about contrasts in different ways, making different predictions. Esthetic appreciation may increase as a consequence of the predictability that comes from repetition, when contrast is minimized. The *prediction effect* posits specifically that fulfilled expectations (i.e., anticipatory successes) lead to positive feelings arising from the limbic reward system (Huron, 2006). From this view, because a contrasting stimulus is unexpected, it should be evaluated unfavorably. Other theorists (Berlyne, 1960, 1971; Eysenck, 1973) propose a trade-off between predictability and novelty as formalized in the *two-factor model* (Berlyne, 1970; Stang, 1974): A stimulus is liked as a function of its arousal potential, which can be too high (e.g., novel) or too low (e.g., predictable). Because the stimulus is evaluated most favorably when it is somewhat familiar but not overly familiar, one would expect increases in liking for music expressing the same emotion after a few exposures, but decreases after many exposures. Finally, high predictability arising from repeated exposure to music expressing the same emotion may lead to habituation or desensitization (i.e., boredom), such that a contrasting or novel stimulus is evaluated favorably (Schubert, 1996).

There are theoretical and empirical reasons for expecting that the third hypothesis could account for listeners’ evaluations of a musical piece that expresses a contrasting emotion, regardless of whether the background (i.e., habituated) emotion is positive or negative. Although positive and negative emotions are typically linked to pleasure and displeasure, respectively, Schubert (1996) suggests that the link between negative emotion and displeasure is de-activated in esthetic contexts that have no consequences for survival, including but not limited to music listening. Any activation in these contexts – positive or negative – is linked to pleasure, such that the *intensity* of the emotional response predicts the degree of pleasure and, hence, the magnitude of the positive appraisal. Moreover, habituation to one type of emotional stimulus should lessen the listener’s arousal level. After sustained or repeated exposure to a single emotion, the expression of another emotion will lead to heightened activation and, consequently, an increase in liking. The increase in liking for the contrasting emotion is primarily a consequence of decreases in liking (habituation) for the sustained or repeated emotion, with the contrasting emotion causing dishabituation and a restoration of activation levels and liking. In the present context, after hearing many, say, happy-sounding pieces of music, listeners should exhibit increased activation and increased liking for a piece of music that expresses a contrasting emotion such as sadness.

Empirical results are consistent with the proposal that increases in emotional activation are predictive of increases in liking for music. Many years ago, Gatewood (1927) observed that pleasure is linked to the intensity of a musical experience rather than the type of experience. In fact, she found that pleasantness ratings were correlated positively with ratings of a variety of different feelings, including sadness, love, longing, amusement, dignity, reverence, how restful the music made listeners feel, or how much the music stirred them. Pleasantness ratings were also correlated with the number of emotions the music activated, and with intensity ratings summed across the different emotions. In another study from the same era (Washburn and Dickinson, 1927), pleasantness ratings were higher for music that evoked feelings of excitement or calmness than for music that evoked a neutral response. In a review of hedonic responses to music and other art forms, Martindale (1984) concluded that esthetic pleasure is typically a positive, monotonic function of emotional activation. More recent research confirms that the intensity of listeners’ emotional responding to music is correlated positively with hedonic ratings (Schubert, 2007a, 2010; Ladinig and Schellenberg, 2012; Vuoskoski et al., 2012).

In an extension of Parducci’s (1995) theory of contextually determined happiness or pleasantness, Huron (2006) described *contrastive valence* as another source of musical pleasure and displeasure. Contrastive valence is based on a mismatch between a musical prediction and the actual outcome. If a positive event is expected, a negative outcome will feel overly unpleasant. By contrast, if a negative event is expected, a positive outcome will feel overly pleasant. In the present study, we sought to extend this line of reasoning to contrasting emotions expressed or evoked by music. If a listener is exposed to several happy-sounding (or sad-sounding) music excerpts in succession, the introduction of a sad-sounding (or happy-sounding) excerpt should sound especially sad (or happy) in contrast. Because listeners’ emotional responses to music tend to parallel the emotions music conveys (Schubert, 2007a,b), especially for happiness and sadness (Hunter et al., 2010), an excerpt that sounds particularly happy or sad because of its contrasting status should evoke a particularly intense emotional response, and consequently greater liking.

Musical pieces differ on many dimensions, which can be continuous (e.g., slow-to-fast, quiet-to-loud) or dichotomous (e.g., major/minor, staccato/legato). Within a single piece, contrasts can occur on a small time scale, such as with alternating consonant or dissonant chords, or on a large time scale, such as with alternations of verse and chorus. Successive movements of a symphony or concerto, or the order of pieces in a concert program represent contrasts on substantially longer time scales. In the present study, we focused on one particular contrast: happiness and sadness. Happy-sounding music tends to be fast in tempo and in major mode, whereas sad-sounding music tends to be slow and minor (for a review see Hunter and Schellenberg, 2010). Happiness and sadness are among the easiest emotions to convey musically (Gabrielsson and Juslin, 1996), particularly when they are contrasted with one another. In fact, young deaf children with cochlear implants – which provide poor spectral resolution and degraded perception of music – can distinguish happy- from sad-sounding music (Volkova et al., 2012).

On each trial in the present experiments, listeners heard a different excerpt of music that sounded unambiguously happy or sad. Results from multiple samples of listeners from the same university population – who listen primarily to dance-pop music (Stalinski and Schellenberg, 2012) – motivated the assumption that excerpts from the particular genre used here (i.e., classical piano pieces) would be unfamiliar to the present listeners. Their task was to rate how much they liked each excerpt and the intensity of their emotional response. Our focus was on responses to excerpts conveying an emotion (e.g., sadness) that contrasted with a background emotion that had been expressed repeatedly (e.g., happiness) with a varying number of presentations.

In Experiment 1, listeners made liking and emotion-intensity ratings in response to 16 different excerpts of music: 14 background excerpts that expressed either happiness or sadness and 2 excerpts that expressed the contrasting emotion. The emotional status of the excerpts had an ABAAAAAAAAAAAAAB order, with A corresponding to the background emotion and B to the contrasting emotion. Thus, the first B excerpt followed a single presentation of an A excerpt, whereas the second B excerpt followed 13 consecutive presentations of different excerpts expressing the A emotion. We predicted that liking and the intensity of listeners’ emotional response would be greater for the second B excerpt than for the immediately preceding A excerpt, whereas responses to the initial A and B excerpts would be similar. This hypothesis applied equally to conditions in which A and B excerpts were happy and sad sounding, respectively, or vice versa.

In Experiment 2, we compared liking and emotion-intensity responses to background and contrasting music excerpts after listeners heard 1, 2, 4, or 8 excerpts that expressed the background emotion. We predicted that as presentation frequency of the background emotion increased, emotion-intensity and liking ratings for the contrasting excerpts would progressively exceed responses to the background excerpts. Because the association between emotional responding and frequency of stimulus presentation tends to be logarithmic (Zajonc, 1968; Harrison, 1977; Bornstein, 1989), we conducted trend analyses to examine effects of presentation frequency, which varied logarithmically.

In both experiments, we predicted that emotional responding (i.e., liking and intensity) to the background excerpts would decrease as the number of presentations increased, whereas responding to the contrasting excerpts would be stable or increase. In other words, we predicted an interaction between emotion type (background or contrasting) and presentation frequency. For both experiments, we predicted that intensity and liking ratings would be positively correlated. Because liking is considered to be a consequence of increases in emotional intensity, we also expected that increases in liking due to emotional contrast would disappear when intensity ratings were held constant.

## Materials and Methods

### Participants

Listeners were undergraduate students enrolled in an introductory psychology course who participated for partial course credit. They were recruited without regard to music training. In Experiment 1, 46 listeners were tested; 29 had taken private music lessons for an average duration of 4.0 years (SD = 3.0 years). When asked about their music-listening habits, only three participants reported listening primarily to classical music, with an additional two indicating that they sometimes listened to classical music. In Experiment 2, 48 new listeners were tested; 26 had taken private music lessons for an average duration of 5.8 years (SD = 4.5 years). Only three participants reported listening primarily to classical music, with an additional three indicating that they sometimes listened to classical music. All participants provided informed written consent, and the experiments were approved by the Office of Research Ethics at the University of Toronto.

### Stimuli

In Experiment 1, stimuli were 28 music excerpts taken from commercially available compact disks, each approximately 30 s in duration (Table 1). In Experiment 2, an additional two excerpts were added to the set. All stimuli were normalized in amplitude to minimize variability in perceived loudness. Stimuli were selected exclusively from nineteenth- and twentieth-century piano music without any vocals or other instruments. Half of the excerpts were selected to convey happiness (major mode, fast tempo); the others were selected to convey sadness (minor mode, slow tempo).

**Table 1:** **Piano recordings used in Experiments 1 and 2**.

Emotion	Title	Composer	Start
Happiness	Excursions, Op.20 – 4. Allegro Molto	Barber	1:24
Happiness	Sonata No16, G major, Op.31 No.1 – 1. Allegro vivace	Beethoven	1:52
Happiness	Sonata No.18, E flat major, Op.31 no.3 – 2. Scherzo Allegretto Vivace	Beethoven	0:45
Happiness	Etude Nr.5 In G Flat, Op. 10, CT 18, Black Keys	Chopin	0:45
Happiness	Etude Nr.8 In F, Op. 10, CT 21	Chopin	0:00
Happiness	Etude Nr.9 In G Flat, Op. 25, CT 34, Butterfly	Chopin	0:00
Happiness	Waltz Nr.4 In F, Op. 34, Valse Brillante	Chopin	0:00
Happiness	Sonata in C, Op.34/1 – 1. Allegro Con Spirito	Clementi	0:00
Happiness	Sonata in C, Op.34/1 – 3. Finale. Allegro	Clementi	0:00
Happiness	Mephisto Waltz Nr. 1	Liszt	0:56
Happiness	Allegro in B Flat, K 400	Mozart	1:20
Happiness	Rondo Nr. 1 in D, K 485	Mozart	0:00
Happiness	Caprices en forme de Valse Op. 2/I. Allegro Moderato	Schumann	0:00
Happiness	IV. Scherzo	Schumann	0:00
Happiness*	Sonata Nr.37, Allegro con Brio	Haydn	0:00
Sadness	Waltz in A Minor, Op.24	Chopin	0:00
Sadness	Sonata Nr. 19 in G Minor, Op.49/1 – 1. Andante	Beethoven	0:00
Sadness	5 Piano pieces, Op.3, Number 3 Largo	R. Strauss	0:00
Sadness	Prelude Nr.4 in E Minor, Op.28	Chopin	0:00
Sadness	Lyric Pieces, Book 4, Op.47 – Melody	Grieg	0:00
Sadness	Fantasia in D Minor, K 397	Mozart	0:00
Sadness	Fantasia in D Minor	Mozart	0:45
Sadness	Fantasy in C Minor, K 475	Mozart	9:35
Sadness	Morceaux de fantaisie op.3 No.1 Elegie in E flat minor	Rachmaninov	0:00
Sadness	Morceaux de fantaisie op.3 No.5 Sérénade in B flat minor	Rachmaninov	1:35
Sadness	Fantasy In F Minor For Piano Duet D.940	Schubert	0:00
Sadness	Variationen uber ein Thema von Robert Schumann Op. 20	Schumann	0:00
Sadness	Trio Romances Op.11 – I. Andante	Schumann	0:00
Sadness	10 Opus 11 No.4	Scriabin	0:00
Sadness*	Sonata Nr.7, Largo e Mesto	Beethoven	0:00

*Used only in Experiment 2.

To verify that the excerpts conveyed the intended emotion, participants judged how happy and how sad each excerpt sounded on five-point scales (1 = *not at all*, 5 = *extremely*) at the end of the test session, after making their liking and emotion-intensity ratings. The excerpts were presented in a different random order for each listener. In Experiment 1, the 14 fast/major excerpts were deemed to sound more happy (*M* = 3.45, SD = 0.79) than sad (*M* = 1.27, SD = 0.36), *t*(45) = 17.70, *p* < 0.001, whereas the 14 slow/minor excerpts were deemed to sound more sad (*M* = 2.99, SD = 0.81) than happy (*M* = 1.54, SD = 0.49), *t*(45) = 10.34, *p* < 0.001. Similarly, in Experiment 2, participants rated the 15 fast/major excerpts as significantly more happy- (*M* = 3.25, SD = 0.66) than sad-sounding (*M* = 1.32, SD = 0.39), *t*(47) = 19.09, *p* < 0.001, and the 15 slow/minor excerpts as more sad- (*M* = 3.02, SD = 0.68) than happy-sounding (*M* = 1.45, SD = 0.47), *t*(47) = 13.04, *p* < 0.001. In both experiments, each individual fast/major excerpt was rated as more happy- than sad-sounding, and each slow/minor excerpt was rated as more sad- than happy-sounding (all *p*s < 0.001). Thus, the stimulus excerpts conveyed the intended emotions.

### Procedure

Listeners were tested individually. They were assigned randomly to one of two conditions, constrained so that happiness was the background emotion for half of them and sadness was the background emotion for the other half. In Experiment 1, each listener heard 16 of the 28 musical excerpts: all 14 that expressed one of the two background emotions, and 2 that expressed the contrasting emotion. In Experiment 2, each listener heard 19 excerpts: all 15 that expressed one of the two background emotions, and 4 that expressed the contrasting emotion. On each trial, listeners rated how much they liked each excerpt (1 = *not at all*, 5 = *extremely*) and the intensity of their emotional response (1 = *felt nothing*, 5 = *highly emotional*). Because the excerpts were selected to sound unambiguously happy or sad, no questions were asked about perceived or felt happiness or sadness until the end of the testing session.

In Experiment 1, the trials began with one presentation of the background emotion (selected randomly) followed by one presentation of the contrasting emotion (selected randomly), followed by 13 excerpts representing the background emotion (in random order) and a second excerpt representing the contrasting emotion (selected randomly). The critical trials of interest involved the two excerpts expressing the contrasting emotion and the two immediately preceding trials that expressed the background emotion (i.e., the first and last presentations of both emotions).

In Experiment 2, excerpts expressing the contrasting emotion occurred after 1, 2, 4, and 8 excerpts that expressed the background emotion. All 24 (i.e., 4!) possible orders of the four presentation frequencies were used. Counterbalanced with condition (happiness or sadness as the background emotion), there were 48 unique presentation orders – one for each of the 48 participants. As in Experiment 1, stimulus selection and order were randomized separately for each listener. Responses to eight critical trials were analyzed: the four that conveyed the contrasting emotion and the four immediately preceding trials that conveyed the background emotion.

## Results

Preliminary analyses confirmed that in both experiments, listeners in the two conditions (happiness vs. sadness as the background emotion) did not differ in terms of gender, age, or years of private music lessons. The principal analyses comprised two mixed-design analyses of variance (ANOVAs): one on liking ratings and the other on emotion-intensity ratings. Both analyses had one between-subjects factor: condition, and two repeated measures: emotion type (background or contrasting) and presentation frequency of the background emotion (Experiment 1: 1 or 13, Experiment 2: 1, 2, 4, or 8).

### Experiment 1

**Figure 1:**
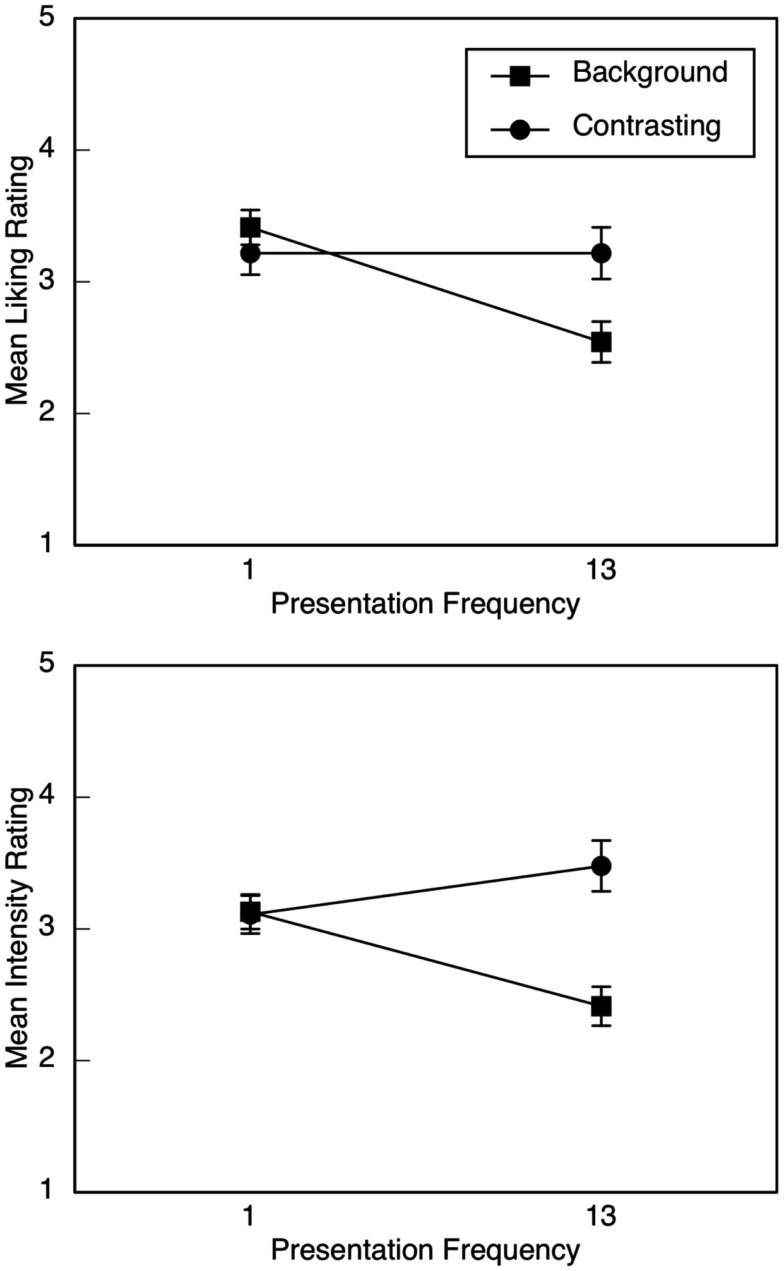
**Mean liking (upper panel) and emotion-intensity (lower panel) ratings in Experiment 1, illustrated as a function of emotion type (background or contrasting) and presentation frequency of the background emotion (1 or 13)**. Error bars are SE.

For liking ratings, there were no main effects or interactions involving condition. Descriptive statistics are illustrated in Figure 1 (upper panel) as a function of emotion type and presentation frequency. A significant interaction between emotion type and frequency, *F*(1, 44) = 15.93, *p* < 0.001, partial η^2^ = 0.27, motivated separate examination of the background and contrasting emotions. Whereas liking ratings for the background emotion declined from the first to the last presentation, *F*(1, 44) = 25.04, *p* < 0.001, partial η^2^ = 0.36, liking ratings for the contrasting emotion were identical, *F* = 0. Moreover, liking ratings did not differ between the background and contrasting emotions during the first two trials, but they did during the final two, *F*(1, 44) = 8.32, *p* = 0.006, partial η^2^ = 0.16, with greater liking for the contrasting emotion. Because there was no three-way interaction, response patterns were similar whether happiness or sadness was the background emotion.

Descriptive statistics for intensity ratings are illustrated in Figure 1 (lower panel) as a function of emotion type and presentation frequency. As with liking ratings, there were no significant effects involving condition. In line with predictions, there was a significant interaction between emotion type and frequency, *F*(1, 44) = 19.42, *p* < 0.001, partial η^2^ = 0.31. For the background emotion, intensity ratings declined from the first to the last presentation, *F*(1, 44) = 13.58, *p* = 0.001, partial η^2^ = 0.24, whereas for the contrasting emotion, intensity ratings increased, *F*(1, 44) = 4.06, *p* = 0.050, partial η^2^ = 0.08. Moreover, intensity ratings did not differ between the background and contrasting emotions during the first two trials, but they did during the final two, *F*(1, 44) = 23.50, *p* < 0.001, partial η^2^ = 0.35, with higher ratings for excerpts expressing the contrasting emotion. As with liking ratings, the lack of a three-way interaction meant that response patterns for intensity ratings were similar whether happiness or sadness was the background emotion.

We calculated correlations between liking and intensity ratings separately for both emotion types (background and contrasting) and both presentation frequencies (1 or 13). Liking and intensity ratings were correlated positively in all four instances (see Table 2). Finally, we repeated the original analysis on liking ratings using multi-level modeling (unstructured covariance matrix) so that intensity ratings could be included as a covariate. Although the association between intensity and liking was highly significant, *F*(1, 151.45) = 122.67, *p* < 0.001, the interaction between emotion type and presentation frequency was eliminated.

**Table 2:** **Correlations between liking and emotion-intensity ratings in Experiments 1 and 2 (all *p*s < 0.05)**.

	Presentation frequency							
	1		2		4		8 or 13	
Exp.	Back	Cont	Back	Cont	Back	Cont	Back	Cont
1	0.32	0.69	–	–	–	–	0.64	0.69
2	0.68	0.75	0.59	0.52	0.55	0.34	0.74	0.49

*Back, background, Cont, contrasting*.

### Experiment 2

**Figure 2:**
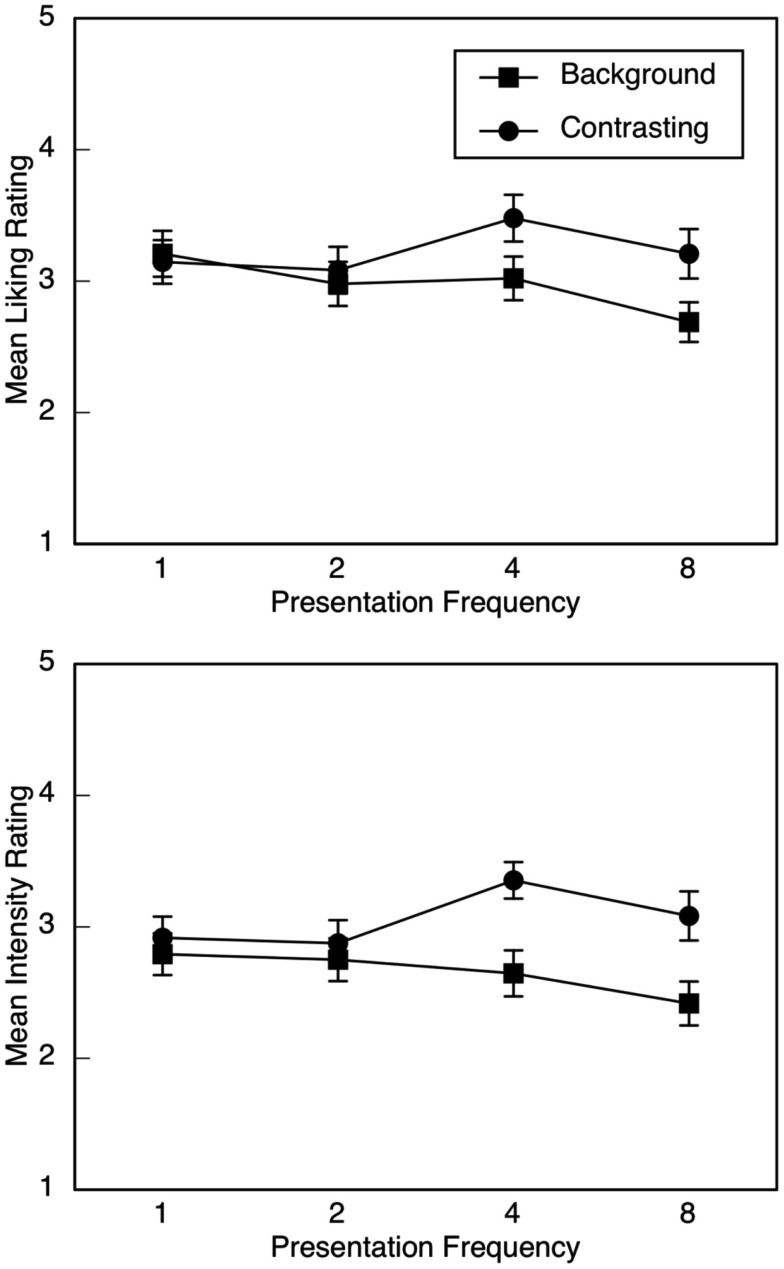
**Mean liking (upper panel) and emotion-intensity (lower panel) ratings in Experiment 2, illustrated as a function of emotion type (background or contrasting) and presentation frequency of the background emotion (1, 2, 4, or 8)**. Error bars are SE.

For liking ratings, there were no main effects or interactions involving condition. Descriptive statistics are illustrated in Figure 2 (upper panel) as a function of emotion type and presentation frequency. As expected, the linear trend for presentation frequency interacted with emotion type, *F*(1, 46) = 9.04, *p* = 0.004, partial η^2^ = 0.16. As the number of presentations increased, liking ratings for the background emotion decreased, *F*(1, 46) = 6.60, *p* = 0.014, partial η^2^ = 0.13, but there was no linear trend for the contrasting emotion. There were no effects involving quadratic or cubic trends. Liking ratings did not differ between the background and the contrasting emotion after one or two presentations, but they approached significance after four presentations, *F*(1, 46) = 3.66, *p* = 0.062, partial η^2^ = 0.07, and differed significantly after eight presentations, *F*(1, 46) = 5.17, *p* = 0.028, partial η^2^ = 0.10.

For intensity ratings, there were again no significant effects involving condition. Descriptive statistics for intensity ratings are illustrated in Figure 2 (lower panel) as a function of emotion type and presentation frequency. As with liking ratings, there was a significant interaction between emotion type and the linear trend for presentation frequency, *F*(1, 46) = 11.98, *p* = 0.001, partial η^2^ = 0.21. As the number of presentations of the background emotion increased, there was a significant decrease in intensity ratings for the background emotion, *F*(1, 46) = 4.08, *p* = 0.049, partial η^2^ = 0.08, but no linear trend for the contrasting emotion. There were no effects involving quadratic or cubic trends. Intensity ratings did not differ between the background and contrasting excerpts after one or two presentations of the background emotion, but they did after four presentations, *F*(1, 46) = 9.46, *p* = 0.004, partial η^2^ = 0.17, and after eight presentations, *F*(1, 46) = 8.16, *p* = 0.006, partial η^2^ = 0.15, with higher ratings for the contrasting excerpt.

As in Experiment 1, liking and intensity ratings were significantly correlated for the background and contrasting excerpts at each of the four presentation frequencies. The eight correlations are presented in Table 2. The final analysis used multi-level modeling on liking ratings, with the same independent variables as in the original mixed-design ANOVA, but with intensity ratings added as a covariate. Although there was a robust association between intensity and liking, *F*(1, 255.81) = 319.48, *p* < 0.001, the interaction between emotion type and the linear trend for presentation frequency disappeared.

## Discussion

The analyses revealed four main findings: (1) listeners reported greater appreciation and a more intense emotional response when the music contrasted in emotional status to that of music heard previously, (2) liking and intensity ratings were correlated positively, (3) the contrast effect for liking disappeared when the intensity of listeners’ emotional responses was held constant, and (4) response patterns were similar whether the background emotion was happiness or sadness.

In line with predictions, both liking and emotion-intensity ratings decreased after hearing many different background excerpts that expressed the same emotion, such that liking and emotion-intensity ratings were larger in comparison for excerpts that expressed a contrasting emotion. Moreover, the results of Experiment 2 provided evidence of a dose-response association: As the frequency of presentation of the background excerpts increased, so did the observed contrast effect. In both experiments, liking and emotion-intensity ratings were correlated, and the contrast effect for liking ratings disappeared when emotional intensity was held constant. Separate randomization for each listener of both excerpt selection and stimulus order ensured that any intrinsic differences in the excerpts’ likeability had no effect on response patterns. Moreover, no stimulus was ever repeated for any participant. Only the emotional character was repeated along with associated features such as mode and approximate tempo. In other words, the results revealed habituation for music on a more abstract level than simple repetition.

In line with Schubert (1996), the observed contrast effect was driven primarily by reductions in emotional responding to music expressing the background emotion as the number of presentations increased. Thus, the effect was mainly a consequence of habituation or desensitization to the background emotion rather than increases in emotional responding to the contrasting emotion. In general, responding to the contrasting emotion remained at baseline levels as presentation frequency of the background emotion increased. The one exception involved emotion-intensity ratings in Experiment 1, which increased above baseline levels for the contrasting emotion after listeners heard 13 different music excerpts that expressed the background emotion. Our documentation of habituation or desensitization to an abstract stimulus property such as emotional character parallels findings from studies of infants that report habituation and/or novelty preferences based on the number of items in a display (e.g., two vs. three; Starkey and Cooper, 1980), categories such as animals (dogs vs. cats; Quinn et al., 1993) or furniture (e.g., chairs vs. couches; Behl-Chadha, 1996), and rules of order with speech sounds (ABB vs. ABA; Marcus et al., 1999).

The present findings serve to inform and evaluate theories relevant to hedonic responding. For example, the two-factor model (Berlyne, 1970; Stang, 1974) fails to account for response patterns because there was no initial increase in liking for music excerpts that expressed the same (background) emotion. In Huron’s (2006) theory of emotional responding to music, the prediction effect posits that pleasure arises from the occurrence of expected events in music, which can be a consequence of simple repetition or variations on a theme (i.e., repetition with subtle changes). Because listeners exhibit greater liking for previously unfamiliar music when they hear it repeatedly in the laboratory, at least up to a point (Meyer, 1903; Getz, 1966; Heingartner and Hall, 1974; Szpunar et al., 2004; Schellenberg et al., 2008), one might expect increases rather than decreases in liking for pieces of music presented sequentially when the pieces express the same emotion. Our results, however, point to *decreases* in liking. As such, the prediction effect may be limited to the positive experience of fulfilled expectancies while listening to a single piece of music, or to repeated presentations of the same piece. Moreover, both theories might be more applicable to a different genre of music (e.g., jazz), timbres other than piano, or to pieces that convey emotions in a more subtle manner.

In the present experiments, listeners’ expectancies or predictions about the emotions expressed by the music excerpts could have worked in two ways. Expectancies for another repetition of the background emotion are consistent with the “hot hand” belief in non-randomness, but different from expectancies for change that are a hallmark of the “gambler’s fallacy” – the false belief that random but independent events are influenced by past occurrences (Burns and Corpus, 2004). For example, after the initial two trials in which all listeners heard one happy- and one sad-sounding excerpt, they may have expected that on subsequent trials, happy- and sad-sounding excerpts would occur equally often, or that the particular emotion an excerpt expressed was determined randomly. Thus, when a contrasting excerpt was presented after a long series of background excerpts, it may have been “overdue” and highly expected or predicted, and therefore pleasurable.

Results from studies of infants show transitions from an initial preference based on stimulus familiarity to one based on novelty (Rose et al., 1982). In the present experiments with adults, relatively rapid habituation to a particular emotion may have been a consequence of the fact that emotions are processed rapidly and automatically (Zajonc, 1980), even when they are expressed musically (Bigand et al., 2005). For example, when an orienting task requires listeners to attend to the emotion expressed by a piece of music, liking for a piece of obviously happy- or sad-sounding music peaks after two exposures (Schellenberg et al., 2008). When the orienting task requires listeners to attend to the lead instrument and the piece is emotionally ambiguous, liking peaks after eight exposures (Szpunar et al., 2004). For different pieces that express the same emotion, only one dimension repeats on the level of the specific emotion (i.e., happiness or sadness). By contrast, a whole piece has many dimensions (e.g., changes in melody, rhythm, harmony, dynamics, and so on), which require more repetitions in order to remember the piece completely. Accordingly, listening to an unfamiliar piece of music initially increases liking for it (Gaudreau and Peretz, 1999), but after many repetitions, liking turns to disliking (Szpunar et al., 2004; Schellenberg et al., 2008).

Our data corroborate and extend Huron’s (2006) notion of contrastive valence, which suggests that a listener’s emotional response is intensified when a musical event contrasts with what is expected. Huron focuses primarily on experiences of pleasantness or unpleasantness that occur in response to unexpected positive and negative musical events, respectively, such that unexpectedness intensifies the listeners’ hedonic evaluation. In the present investigation, listeners reported more intense responses to music whose emotional character contrasted with music heard previously, which, in turn, led to relatively positive evaluations whether the music was happy or sad sounding. Note that the effect size of the crucial interaction (i.e., between presentation frequency and emotion type) was larger for emotion-intensity than for liking ratings in both experiments (compare the upper and lower panels in Figures 1 and 2), an additional finding consistent with our hypothesis that the intensity of the emotional response would determine the evaluative response. Moreover, in Experiment 2, after four presentations of the background emotion, differences between the background and contrasting excerpts were significant for intensity ratings but only marginal for liking ratings.

Can we be certain that listeners were actually responding emotionally to the excerpts rather than simply perceiving the emotions conveyed? We know that music listeners reliably distinguish the two types of responses when asked to rate their feelings *and* perceptions (Kallinen and Ravaja, 2006; Schubert, 2007b; Evans and Schubert, 2008; Hunter et al., 2010). In the present study, listeners were told specifically to rate the intensity of their feelings, not the intensity of the emotions conveyed by the excerpts, and we have no reason to doubt that they followed instructions. In any event, because perception and feeling ratings in response to music tend to vary in tandem (Kallinen and Ravaja, 2006; Hunter et al., 2010), with feelings mediated by perceptions in some circumstances (Hunter et al., 2010), feelings are difficult to tease apart from perceptions, which almost certainly played a role in the observed response patterns. For example, if listeners had been required to rate the happiness and sadness expressed by the excerpts during (instead of after) the actual test phase, we are confident that a perceptual contrast effect would have emerged, as it has in previous studies of perceived lightness, loudness, or temperature.

Our findings are also consistent with Schubert’s (1996) proposal that the intensity of the emotional response predicts the degree of pleasure and, consequently, the magnitude of the positive appraisal. Schubert’s theory further suggests that music deemed sad is enjoyed because the link between negative emotions and displeasure is de-activated in esthetic contexts. Huron (2006) expanded on this suggestion by proposing that the mechanism for increased liking of a contrasting musical stimulus is (slow) cortical inhibition of (fast and automatic) subcortical responses. In the end, the cognitive appraisal inevitably concludes that nothing bad has occurred, and that one is simply listening to sad-sounding music. The results of our experiments contribute to a longstanding paradox that has intrigued both esthetic philosophers as well as psychologists – why listeners often enjoy sad-sounding music (Robinson, 1994; Davies, 2003; Schellenberg et al., 2008; Garrido and Schubert, 2010, 2011; Hunter et al., 2011; Van den Tol and Edwards, 2011; Ladinig and Schellenberg, 2012; Vuoskoski and Eerola, 2012; Vuoskoski et al., 2012).

Although listeners tested in the laboratory generally prefer happy- over sad-sounding music (Thompson et al., 2001; Husain et al., 2002; Gosselin et al., 2005; Hunter et al., 2008, 2010), this preference can be eliminated when the listeners are fatigued (Schellenberg et al., 2008) or in a sad mood (Hunter et al., 2011). In other words, negative psychological states can motivate listening to sad-sounding music (Van den Tol and Edwards, 2011). Liking sad-sounding music is also correlated with individual differences in personality – positively with openness-to-experience, empathy, and absorption, but negatively with extraversion (Garrido and Schubert, 2011; Ladinig and Schellenberg, 2012; Vuoskoski et al., 2012). The present findings highlight another contextual factor associated with increased appreciation of sad-sounding music: repeated exposure to happy-sounding music. Our results also provide a cultural-level explanation for choosing to listen to sad-sounding music, or at least to sad-sounding classical music. Because the majority of such music sounds relatively happy (i.e., fast tempo and major mode; Post and Huron, 2009), listeners may enjoy sad-sounding music simply because of its relative rarity – and hence contrast – in a culture in which happy-sounding music is more prevalent.

Our findings raise additional questions that could be addressed in future research. For example, on each trial of the present experiments, listeners attended closely to the music because they were required to provide ratings of how much they liked each excerpt and the intensity of their emotional response. Although such focused listening is common in some contexts (e.g., while attending a concert), the majority of day-to-day listening involves music heard incidentally while listeners are performing some other task (Sloboda et al., 2001). Moreover, 32 presentations of incidental music leads to progressively higher liking ratings (Szpunar et al., 2004; Schellenberg et al., 2008), which raises the possibility that the contrast effects observed here would not extend to incidental listening. In principle, repetition of different excerpts expressing the same emotion could lead to *higher* liking ratings.

Another potential avenue for future research would be to substitute self-reports of emotion-intensity with measures of physiological changes in arousal (e.g., skin conductance or heart rate), which would provide objective indicators of the intensity of the listener’s emotional response. Stimulus selection is also bound to play a role in the contrast effects we observed. The present studies made use of excerpts from classical piano music, a style of music unlikely to be favored by Canadian undergraduates. It remains unknown whether the contrast effect would be stronger or weaker with more familiar and/or well-liked styles of music. In one study, a preference for classical music was associated with more intense emotional responding to such music (Kreutz et al., 2008). The limits of the role of the intensity of the listener’s emotional response could also be tested. An intense but negative emotional response (e.g., aversion evoked by misogynistic hip-hop lyrics or extremely dissonant music) is unlikely to be accompanied by increases in liking. Finally, interaction effects with mood are likely to be evident. Sad-sounding music evokes sad moods (Hunter et al., 2008, 2010; Vuoskoski and Eerola, 2012), and listeners in negative moods show increased liking for sad-sounding music (Schellenberg et al., 2008; Hunter et al., 2011; Van den Tol and Edwards, 2011). Thus, in some contexts, one might observe *increased* liking for a sad-sounding musical piece after listening to other sad-sounding pieces.

In summary, our results reveal that when listeners attend closely to different pieces of music, they progressively habituate to music that maintains the same emotional character. Hence, they show greater appreciation for music that conveys a contrasting emotion. Such contrast effects appear to occur because repeatedly conveying the same emotion dulls the listener’s emotional response, whereas conveying a contrasting emotion intensifies the response. Music composers are likely to be aware of this contrast effect, either implicitly or explicitly, by using contrasting musical characteristics (e.g., tempo, mode, and dynamics) to increase the intensity of listeners’ emotional response and their liking of different sections of a particular composition, or of successive compositions on an album. Moreover, similar contrast effects are likely to be evident in other art forms, such as dance, theater, and visual art. Our results highlight the importance of emotional responding in hedonic evaluations and raise new questions about the role of contrasts in esthetic appreciation.

## Conflict of Interest Statement

The authors declare that the research was conducted in the absence of any commercial or financial relationships that could be construed as a potential conflict of interest.
